# Hospital onset bacteremia and fungemia should be a pay-for performance measure: a pro/con debate

**DOI:** 10.1017/ice.2025.10216

**Published:** 2025-09

**Authors:** Gregory M. Schrank, Theresa Madaline

**Affiliations:** 1 Department of Medicine, University of Maryland School of Medicine, Baltimore, MD, USA; 2 Department of Medicine, Columbia University Vagelos College of Physicians and Surgeons, New York, NY, USA

## Abstract

Healthcare-associated infections (HAIs) result in substantial patient harm and avoidable costs. Pay-for-performance programs (PFP) through the Centers for Medicare and Medicaid Services (CMS) have resulted in reductions of HAIs like central line-associated bloodstream infections (CLABSI) and methicillin-resistant *Staphylococcus aureus* bacteremia, through robust infection prevention programs and practices. Hospital Onset Bacteremia and Fungemia (HOB) is proposed as an alternative quality measure for public reporting and PFP, and was endorsed by the National Quality Forum in 2022. This broad measure is designed as an electronic quality measure that avoids manual abstraction and excludes risk adjustment. HOB would substantially expand the scope of focus of existing bloodstream infection measurement, and is currently being considered for voluntary reporting in 2025. In this article, we provide arguments for and against adopting HOB as a PFP measure linked to CMS payments.

## Introduction

Due to the patient harm sustained from healthcare-associated infections (HAIs), as well as the substantial associated economic costs, the Institute of Medicine issued a 2006 report that called for a pay-for-performance (PFP) mechanism within Medicare that “can help transform the payment system into one that rewards both higher value and better outcomes”.^1–3^ This policy strategy - to reward performance and disincentivize low value or harmful care - was integrated into value-based programs, like the Hospital-Acquired Condition Reduction Program, by the Centers for Medicare and Medicaid Services (CMS), and penalizes hospitals that are low performing against a number of publicly reportable HAIs by withholding payments.^4^ The introduction of this program fundamentally transformed HAIs from clinical diagnoses to standard measures of quality across US hospitals, and drove facilities to develop robust infection prevention programs focused on improving patient care. Simultaneously, these PFP measures became levers of control, front and center in hospital board rooms, with algorithmic surveillance protocols and significant implications for the financial and reputational health of healthcare facilities.

Central line-associated bloodstream infections (CLABSIs), a core measure of CMS’ HAC and value-based payment programs, is a particularly notable measure given the high morbidity associated with the diagnosis in comparison with other HAIs **(**Table 1). Since the inclusion of CLABSI as a reportable, PFP measure, the incidence has markedly decreased, representing a clear success for patient safety over the past two decades.^5,6^ In large part due to these reductions, CLABSIs currently represent a minority of nosocomial bloodstream infections – estimated around 20–30% - and an even lower proportion among neonates.^7–9^ In 2013, methicillin-resistant Staphylococcus aureus bloodstream infections (MRSA BSI) were added to mandatory reporting, ultimately resulting in further decreases in nosocomial bloodstream infection events.^10^ Hospital Onset Bacteremia and Fungemia (HOB) is proposed as an alternative quality measure for public reporting and PFP, and was endorsed by the National Quality Forum in 2022.^11^ Defined as a blood culture collected on hospital day ≥4 with growth of a pathogenic bacteria or fungi, HOB would substantially expand the scope of focus of existing bloodstream infection measurement, and is currently being considered for voluntary reporting in 2025.^12,13^ In this article, we provide the arguments for and against adopting HOB as a PFP measure linked to CMS payments.

**Table 1.** Comparison of impact of hospital-onset bacteremia and selected existing hospital-acquired infection measures

	HOB	CLABSI	MRSA Bacteremia	CAUTI	SSI
Annual Incidence (cases/year)	102,361–120,009^23^	23,389^54^	9,830^54^	20,237^54^	22,416^54^
Mortality (relative risk)	3.20^55^	3.77^55^	3.5^56^	1.50^57^	3.32^57^
Excess LOS (days)	13.8^55^	16.8^55^	9.2^56^	0.63^58^	11.2^55^
Attributable Cost per case (USD)	$35,310^55^	$49,400^55^	$30,998^59^	$13,793^57^	$28,219^57^
Preventability	41%^7^	65%^60^	80%^61^	70%^60^	55%^60^

HOB, hospital-onset bacteremia; CLABSI, central line-associated bloodstream infection; MRSA, methicillin-resistant *Staphylococcus aureus*; CAUTI, catheter-associated urinary tract infection; SSI, surgical site infection; LOS, length of stay

## Hospital Onset Bacteremia and Fungemia as a Pay-For Performance Measure: Pro

### HOB vs existing measures

When considering the adoption of HOB as a PFP measure of bloodstream infections, it is first important to acknowledge the limitations of CLABSI for this same purpose. CLABSIs, by definition, are limited to bloodstream infection events contemporaneous with indwelling central venous catheters.^14^ While this is intuitive when trying to identify patient harm related to the device, it presents a challenge of feasibility for surveillance given the difficulty of establishing a causal relationship between the presence of the catheter and the bloodstream infection (BSI). Thus, the process of hospitals determining and reporting CLABSI events relies upon teams of individuals, often infection preventionists, applying complex NHSN rules and algorithms and often working backwards to exclude other sources of BSI first before “settling on” a CLABSI.^15,16^ The process followed is heterogeneous across hospital facilities, filled with inaccuracy due to inexperienced case reviewers and subjectivity in assessment. Given the financial and reputational stakes, it is prone to immense bias. Indeed, multiple statewide studies demonstrated marked underreporting of CLABSI events.^16–18^ A survey of the SHEA Research Network found that the vast majority of hospital epidemiologists believe that current HAI quality measures are susceptible to gaming and manipulation, including CLABSI.^19^

For cases that are actually reported, the incidence of CLABSI has declined to levels that limit meaningful comparison between hospitals, the entire purpose of the CMS Hospital Compare Program.^20^ In addition, the lack of automation in this review process requires substantial investment of time, often by infection preventionists or hospital quality/patient safety teams, detracting from other work; it’s estimated that HAI surveillance occupies about one quarter of time spent by infection preventionists.^19,21,22^ The cumbersome nature of CLABSI as a measure, along with its intrinsically biased reporting system, underlies the push for a better alternative.

### Impact of a HOB performance measure

Weighing the merits of HOB as a PFP measure, one central tenet is that nosocomial bloodstream infections are a cause of substantial morbidity, mortality, and excess healthcare utilization in the United States (Table 1). Estimates of mortality associated with HOB events range from 15–30%.^23^ From the perspective of hospitals and payors, HOB events are associated with excess cost due to increased hospital length of stay and readmission rates.^7,23^ These cost estimates do not include an analysis of the financial harm to patients who bear the burden of additional costs.^24^ By adopting HOB as a PFP measure, focus on quality and cost containment will expand beyond the devices and surgical procedures of current HAI metrics, and the commonality of these infection events will facilitate more effective comparison of quality between facilities. Research to date has shown that HOB events arise from a diverse range of sources; most commonly gastrointestinal, endovascular, and the respiratory tract.^7^ Leekha et al. demonstrated that less than half of hospital-onset bloodstream infection events due to non-commensal organisms are from sources that are included in current routine surveillance for PFP measures, and their prevention is thereby de-prioritized.^7,12^

The study by Leekha et al. also demonstrated that expansion from CLABSI to HOB adds surveillance for a substantial proportion of preventable nosocomial BSI events that are thereby actionable for improvement efforts. Using an expert panel-validated HOB preventability rating guide, a study of almost 1800 non-commensal HOB events across 13 hospitals found that among HOB events with non-commensal organisms, 36% were considered preventable.^7^ Of these preventable HOB events, 39% are currently not included in existing routine surveillance.^7^ Peripheral IV (PIV) catheter-associated thrombophlebitis is a key example of BSIs not included in current surveillance. The infection risk of each PIV is very low, but due to high exposure from an enormous number of PIV catheters placed each year in the US, they are estimated to contribute to about half of all catheter-related *Staphylococcus aureus* bacteremia events.^25,26^ Additionally, a study of real world HOB events at six hospitals utilized root cause analysis case reviews to identify factors contributing to these infections and identified opportunities for quality improvement across a broad range of domains including systems and human factors.^27^ There is a clear need, and yet, there is currently no mechanism for systematic surveillance or structure to incentivize prevention of these infections.

Much of the ongoing work by hospitals to prevent CLABSIs and MRSA bloodstream infections can immediately be applied to HOB prevention efforts, so hospitals will not need to completely shift strategies.^28,29^ However, the expansion of surveillance to include sources of infection beyond specific devices and a limited number of surgical procedures will inevitably challenge hospital infection prevention and quality teams due to the high frequency of the events and the lack of clear prevention strategies for all sources of infection. It is exactly this challenge that will drive innovative means of reducing harm among hospitalized patients. Whether it be through improved blood culture diagnostic stewardship efforts, optimizing strategies of pathogen bioburden reduction, or even supplementing surveillance and patient safety with emerging artificial intelligence models, HOB as a PFP measure will spark a paradigm shift in HAI prevention, for the benefit of patients and health systems alike.^12,30^

### Measurement and reporting

For HOB to be successful as a quality measure, careful consideration is needed to define inclusion and exclusion criteria to best measure preventable harm, reflect healthcare quality, and avoid excess penalization due to imbalances in risk incidence across facilities. In the aforementioned study, Leekha et al. identified characteristics of HOB events that are of very low preventability—most notably neutropenia—which can be applied as exclusion criteria for a PFP measure. Additional research has already provided the ground work for appropriate risk adjustment, including benchmarking of blood culture utilization, electronically available comorbidities, the preventability of various HOB sources, and socioeconomic determinants of health outcomes that are critical for inclusion to avoid over-penalization of academic and safety-net hospitals.^31–34^

One of the greatest strengths of HOB as a quality measure is its simplicity. Requiring only time from admission and blood culture results, HOB events can be easily identified without the need for manual review and adjudication and are amenable to automation. HOB is under development as one of the CDC’s first digital quality measures, as part of its broader Data Modernization Initiative.^35^ Utilizing a platform known as Fast Healthcare Interoperability Resources—or FHIR - this automation will facilitate more real-time surveillance and data collection at the patient level. Outside of the US, scaled automation was already demonstrated across a multi-year period in four Dutch hospitals.^36^ Automated data transfer will allow the application of exclusion rules or risk adjustment to be performed after data collection, thereby liberating hospital-based teams from the labor and bias inherent to CLABSI surveillance.^35^

### Financial incentive

While the expansion of HAI surveillance and public reporting helps to understand the scope of the problem, linkage to payment will provide the incentive for improvement. Once CMS enacted its HAC program for specific HAIs in 2008, there was an observed reduction in CLABSI and CAUTI rates over the subsequent two years, as well a modest reduction in orthopedic surgical site infections which was observed across all patients, not just Medicare recipients for whom the payment penalties apply.^37–39^ Current financial health measures of hospitals, with razor-thin operating margins, imply that payment incentives will continue to be effective methods of driving change.^40^ And while not all PFP measures have been effective or come without unintended consequences (e.g., the impact of the SEP-1 bundle on broad-spectrum antibiotic prescribing), the objectivity of the HOB outcome along with appropriate risk adjustment will mitigate detrimental effects.^41–44^

## Hospital Onset Bacteremia and Fungemia as a Pay-For Performance Measure: Con

### HOB vs existing measures

Mandatory CLABSI reporting has had a positive impact on clinical outcomes and appropriate catheter utilization. Transition to reporting HOB will dilute the focus on appropriateness of vascular access and associated harms including infection, thrombosis, vascular injury, extravasation, pain, and inappropriate blood sampling. The serious setbacks in CLABSI reduction as a result of the COVID-19 pandemic are a clear indication that shifting the focus away from existing HAI measures and reporting can have rapid and significant patient safety consequences. The CDC suspended reporting requirements in the early months of the pandemic; when reporting resumed, there was a dramatic 45% increase in the standardized infection ratio nationally in the first quarter of 2021^61^. This strongly suggests that a decrease in surveillance, staffing challenges, and clinical practice changes can quickly result in an increase in CLABSI events. Retiring CLABSI reporting in favor of HOB could erode years of progress in central venous catheter-related harm reduction.

### Impact of a HOB performance measure

There is no existing evidence that implementing a HOB measure leads to better patient outcomes when added to existing HAI programs. The goal of HAI reporting programs is to improve care through specific prevention strategies. The National Quality Forum Hospital-Onset Bacteremia and Fungemia Playbook directs institutions to perform a literature search to identify HOB best practices, find gaps between current state and evidence-based practice, and compare current performance against existing benchmarks.^45^ However, no implementation literature, evidence-based guidelines, or benchmarks for HOB exist. This begs the question of how a more generalized HAI measure without accompanying strategies or prevention tools will drive better outcomes. NQF directs institutions to use existing HAI bundles, but these offer little benefit beyond existing ongoing HAI prevention activities and suggest HOB measurement would result in limited real-world impact.

Only a fraction of HOB events are preventable; potentially a lower proportion than other current HAI measures (Table 1).^7^ Leekha et. al. demonstrated that of all HOB events, gastrointestinal source was most frequent (35%), of which a third were due to mucosal barrier injury.^7^ These events were considered by reviewers to have low preventability. Furthermore, the second most common source was endovascular (32%); of these, 77% were CLABSI that would have been captured by current reporting and 23% were due to *Staphylococcus aureus*, another existing HAI measure. In their sensitivity analysis, Leekha et. al. identify only 19% of non-commensal HOB events that were considered very likely preventable by reviewers. On multivariate analysis, non-commensal HOB events were considered more likely to be preventable if originating from intravascular catheters, indwelling urinary catheters, or surgical site infection; most of these events are already captured by existing HAI reporting. Conversely, neutropenia, immunosuppression, gastrointestinal, bone/joint, or unidentified sources had a lower likelihood of being rated preventable. These risk factors will vary greatly across institutions and populations, and there is no proposed mechanism to capture or adjust for these factors in the new HOB measure. Together, these limitations make the HOB measure poorly actionable and comparable across institutions and care settings.

HOB is designed to be a dCQM that will require less infection preventionist effort and time than current HAI measures. As designed, however, there is limited opportunity to analyze additional information on preventability, risk factors, and source to create actionable data. The NQF HOB playbook acknowledges organizations will need to collect and track a host of other data elements to make the HOB measure meaningful and suggests training a data coordinator with specialty training, like an infection preventionist.^45^ Consequently, the adoption of HOB may not result in infection preventionists having less surveillance-based work or more time to focus on supporting healthcare teams.

### Measurement and reporting

Bacteremia from a wide variety of primary sources can occur in or outside of the healthcare environment with serious clinical consequences. While some events in the healthcare setting are clearly iatrogenic and represent opportunities for improvement, others are clearly a result of an underlying disease process and prevention is unlikely. It is well-established that existing HAI measures like CLABSI have limitations such as attribution, true preventability and over-diagnosis.^46^ The same limitations exist for HOB, but to a greater extent in the absence of any risk adjustment strategy or attribution assessment. Adoption of a non-risk adjusted HOB measure is likely to disincentivize providers from accepting HOB as a true measure of preventable harm worthy of improvement. Furthermore, existing CLABSI criteria fail to adequately adjust for certain patient populations, clinical scenarios, and level of care (gastrointestinal sources, immunosuppression, intra-aortic balloon pumps, patient-related manipulation or refusal of maintenance, extremely premature newborns, stepdown level of care). If the current CLABSI definition and risk adjustment fail to account for these factors, expanding measurement to all bacteremia and fungemia events with no risk adjustment would inevitably lead to conflict and poor engagement.

Institutions that provide long-term acute care, subacute rehabilitation, or care for other chronically ill populations or the under-insured are likely to be disproportionately impacted by HOB reporting in the absence of risk adjustment for comorbid conditions. Prior studies have shown that PFP HAI programs disproportionately penalize safety net hospitals rather than funding additional prevention resources to address their unique needs.^47^ HOB will worsen existing disparities for these facilities rather than supporting HAI prevention efforts.

Seasonal effects and climate-related factors are associated with increases in bacterial and fungal bloodstream infections.^48^ Respiratory viral illness (e.g. COVID-19, influenza) is associated with bacterial superinfection, and incidence of viral illness is often seasonal or cyclical.^49,50^ Without adjustment for or consideration of respiratory illness patterns and other seasonal changes, there will be temporal and geographic disparities in HOB rates.

### Financial incentive

Existing PFP programs have resulted in controversial strategies to avoid penalties associated with HAI, including CLABSI.^51^ Similar to questionable strategies employed to reduce CLABSI rates, reduction of HOB rates by avoiding blood cultures altogether, obtaining low volume of blood, or initiation of empiric broad antimicrobials prior to sending blood cultures is likely. We may see a drop in blood cultures acquired and a concurrent rise in empiric prescribing without an effective mechanism to account for blood culture frequency. This poses a serious risk of patient harm and antimicrobial resistance (AMR) including increased incidence of multidrug-resistant organisms, *C. difficile* infection, and delayed identification and appropriate treatment of pathogens.

It is important to learn from previous unsuccessful infection-related metrics, such as pneumonia and sepsis, and the resulting unintended consequences for antimicrobial stewardship and AMR reduction efforts.^52,53^ While antimicrobial utilization (AU) reporting has been suggested as a mechanism to prevent inappropriate antimicrobial prescribing after HOB implementation, this is unlikely to be the case. AU benchmarking is not available and is not a part of any PFP programs; therefore, it is unlikely to deter hospitals from over-utilizing antimicrobials in response to HOB implementation.

## Recommendations

A HOB measure has the potential to enhance safety and develop new improvement strategies for preventable HOB events. However, to achieve this goal, we recommend several adjustments to the measure. Risk adjustment for patient factors and exclusion of low-preventability events will be critical for engagement. Blood culture utilization should be included as a required balancing measure with HOB reporting. Both HOB and blood culture utilization benchmarks should be determined after an appropriate period of measure reporting; such benchmarks must be established before HOB can be considered as a pay-for-performance measure. CLABSI should not be considered for retirement until more is known about HOB benchmarking and impact. HOB-specific toolkits and improvement strategies will be required to successfully support institutions in reducing HOB incidence; funds allocated to HOB prevention implementation science would be beneficial in establishing best practices and prevention tools.

## References

[1] Douglas S. The Direct medical costs of healthcare-associated infections in U.S. hospitals and the benefits of prevention. Division of Healthcare Quality Promotion, Centers for Disease Control and Prevention; 2009.

[2] Roberts RR , Scott RD, 2nd, Hota B , et al. Costs attributable to healthcare-acquired infection in hospitalized adults and a comparison of economic methods. Med Care 2010;48:1026–1035 20940650 10.1097/MLR.0b013e3181ef60a2

[3] Committee on Redesigning Health Insurance Performance Measures, Payment, and Performance Improvement Programs. Rewarding Provider Performance: Aligning Incentives in Medicare. Institute of Medicine of The National Acadamies. Washington, D.C.: The National Academies Press.

[4] Hospital-Acquired Condition Reduction Program. 2024. https://www.cms.gov/medicare/payment/prospective-payment-systems/acute-inpatient-pps/hospital-acquired-condition-reduction-program-hacrp.

[5] Magill SS , O’Leary E , Janelle SJ , et al. Changes in prevalence of health care-associated infections in U.S. Hospitals. N Engl J Med 2018;379:1732–1744 30380384 10.1056/NEJMoa1801550 PMC7978499

[6] National Center for Emerging and Zoonotic Infectious Diseases DoHQP. 2023 National and State Healthcare-Associated Infections Progress Report. https://www.cdc.gov/healthcare-associated-infections/php/data/progress-report.html.

[7] Leekha S , Robinson GL , Jacob JT , et al. Evaluation of hospital-onset bacteraemia and fungaemia in the USA as a potential healthcare quality measure: a cross-sectional study. BMJ Qual Saf 2024;33:487–498 10.1136/bmjqs-2023-016831 PMC11287649 38782579

[8] Rock C , Thom KA , Harris AD , et al. A Multicenter Longitudinal Study of Hospital-Onset Bacteremia: Time for a New Quality Outcome Measure? Infect Control Hosp Epidemiol 2016;37:143–148 26493213 10.1017/ice.2015.261 PMC4731295

[9] Prochaska EC , Xiao S , Colantuoni E , et al. Hospital-Onset Bacteremia Among Neonatal Intensive Care Unit Patients. JAMA Pediatr 2024;178:792–799 38913368 10.1001/jamapediatrics.2024.1840 PMC11197452

[10] Centers for Disease Control and Prevention. 2023 National and State Healthcare-Associated Infections Progress Report. 2024. https://www.cdc.gov/healthcare-associated-infections/php/data/progress-report.html#cdc_report_pub_study_section_2-data-highlights.

[11] Partnership for Quality Measurement. https://p4qm.org/measures/3686.

[12] Schrank GM , Snyder GM , Leekha S. Hospital-onset bacteremia and fungemia: examining healthcare-associated infections prevention through a wider lens. Antimicrob Steward Healthc Epidemiol 2023;3:e198 38028924 10.1017/ash.2023.486 PMC10654956

[13] Betz KDRS , N.; Stutler , L. NHSN’s New Digital Quality Measures: Introduction and Overview. National Center for Emerging and Zoonotic Infectious Diseases, Centers for Disease Control and Prevention, 2024.

[14] Central Line-associated Bloodstream Infection (CLABSI) Basics. Centers for Disease Control and Prevention. 2024. https://www.cdc.gov/clabsi/about/index.html.

[15] National Healthcare Safety Network. Bloodstream Infection Event (Central Line-Associated Bloodstream Infection and Non-central Line Associated Bloodstream Infection). 2025. https://www.cdc.gov/nhsn/pdfs/pscmanual/4psc_clabscurrent.pdf.

[16] Sexton DJ , Chen LF , Moehring R , Thacker PA , Anderson DJ. Casablanca redux: we are shocked that public reporting of rates of central line-associated bloodstream infections are inaccurate. Infect Control Hosp Epidemiol 2012;33:932–935 22869268 10.1086/667383

[17] Lin MY , Hota B , Khan YM , et al. Quality of traditional surveillance for public reporting of nosocomial bloodstream infection rates. JAMA 2010;304:2035–2041 21063013 10.1001/jama.2010.1637 PMC8385387

[18] Mayer J , Greene T , Howell J , et al. Agreement in classifying bloodstream infections among multiple reviewers conducting surveillance. Clin Infect Dis 2012;55:364–370 22539665 10.1093/cid/cis410

[19] Schrank GM , Branch-Elliman W , Leekha S , et al. Perceptions of Health Care-Associated Infection Metrics by Infection Control Experts. JAMA Netw Open 2023;6:e238952 37074719 10.1001/jamanetworkopen.2023.8952 PMC10116362

[20] Centers for Medicare and Medicaid Services. Hospital Quality Initiative Public Reporting: Hospital Care Compare and Provider Data Catalog. https://www.cms.gov/medicare/quality/initiatives/hospital-quality-initiative/hospital-compare.

[21] Pogorzelska-Maziarz M , de Cordova PB , Herzig CTA , Dick A , Reagan J , Stone PW. Perceived impact of state-mandated reporting on infection prevention and control departments. Am J Infect Control 2019;47:118–122 30322814 10.1016/j.ajic.2018.08.012 PMC6359958

[22] Landers T , Davis J , Crist K , Malik C. APIC MegaSurvey: Methodology and overview. Am J Infect Control 2017;45:584–588 28126260 10.1016/j.ajic.2016.12.012

[23] Goto M , Al-Hasan MN. Overall burden of bloodstream infection and nosocomial bloodstream infection in North America and Europe. Clin Microbiol Infect 2013;19:501–509 23473333 10.1111/1469-0691.12195

[24] Coomer NM , Kandilov AM. Impact of hospital-acquired conditions on financial liabilities for Medicare patients. Am J Infect Control 2016;44:1326–1334 27174461 10.1016/j.ajic.2016.03.025

[25] Swaminathan L , Flanders S , Horowitz J , Zhang Q , O’Malley M , Chopra V. Safety and Outcomes of Midline Catheters vs Peripherally Inserted Central Catheters for Patients With Short-term Indications: A Multicenter Study. JAMA Intern Med 2022;182:50–58 34842905 10.1001/jamainternmed.2021.6844 PMC8630646

[26] Mermel LA. Short-term Peripheral Venous Catheter-Related Bloodstream Infections: A Systematic Review. Clin Infect Dis 2017;65:1757–1762 29020252 10.1093/cid/cix562

[27] Schrank GM , Leekha S , Brady M , et al. P-407. Evaluation of Use of Hospital-onset Bacteremia and Fungemia (HOB) for Quality Improvement. Open Forum Infectious Diseases 2025;12

[28] Leekha S , Li S , Thom KA , et al. Comparison of total hospital-acquired bloodstream infections to central line-associated bloodstream infections and implications for outcome measures in infection control. Infect Control Hosp Epidemiol 2013;34:984–986 23917916 10.1086/671730 PMC4070376

[29] Dantes RB , Abbo LM , Anderson D , et al. Hospital epidemiologists’ and infection preventionists’ opinions regarding hospital-onset bacteremia and fungemia as a potential healthcare-associated infection metric. Infect Control Hosp Epidemiol 2019;40:536–540 30932802 10.1017/ice.2019.40 PMC6897303

[30] Rodriguez-Nava G , Egoryan G , Goodman KE , Morgan DJ , Salinas JL. Performance of a large language model for identifying central line-associated bloodstream infections (CLABSI) using real clinical notes. Infect Control Hosp Epidemiol 2024;46:1–4 10.1017/ice.2024.164 PMC11883649 39473230

[31] Serpa JA , Gemeinhardt G , Arias CA , et al. Teaching and Safety-Net Hospital Penalization in the Hospital-Acquired Condition Reduction Program. JAMA Netw Open 2024;7:e2356196 38363569 10.1001/jamanetworkopen.2023.56196 PMC10873765

[32] Fabre V , Hsu YJ , Carroll KC , et al. Blood Culture Use in Medical and Surgical Intensive Care Units and Wards. JAMA Netw Open 2025;8:e2454738 39813030 10.1001/jamanetworkopen.2024.54738 PMC11736503

[33] Yu KC , Ye G , Edwards JR , et al. Hospital-onset bacteremia and fungemia: An evaluation of predictors and feasibility of benchmarking comparing two risk-adjusted models among 267 hospitals. Infect Control Hosp Epidemiol 2022;43:1317–1325 36082774 10.1017/ice.2022.211 PMC9588439

[34] Jackson SS , Leekha S , Magder LS , et al. The Effect of Adding Comorbidities to Current Centers for Disease Control and Prevention Central-Line-Associated Bloodstream Infection Risk-Adjustment Methodology. Infect Control Hosp Epidemiol 2017;38:1019–1024 28669363 10.1017/ice.2017.129 PMC5711399

[35] Centers for Disease Control and Prevention. NHSN Digital Quality Measures (dQMs). 2024. https://www.cdc.gov/nhsn/fhirportal/index.html.

[36] Brekelmans M , Vlek ALM , van Dijk Y , et al. Automated surveillance of hospital-onset bacteremia and fungemia: feasibility and epidemiological results from a Dutch multicenter study. Infect Control Hosp Epidemiol 2015;175:347–354 10.1017/ice.2025.29 PMC12034453 40007429

[37] Waters TM , Daniels MJ , Bazzoli GJ , et al. Effect of Medicare’s nonpayment for Hospital-Acquired Conditions: lessons for future policy. JAMA Intern Med 2015;175:347–354 25559166 10.1001/jamainternmed.2014.5486 PMC5508870

[38] Kwong JZ , Weng Y , Finnegan M , et al. Effect of Medicare’s Nonpayment Policy on Surgical Site Infections Following Orthopedic Procedures. Infect Control Hosp Epidemiol 2017;38:817–822 28487001 10.1017/ice.2017.86

[39] Kim KM , White JS , Max W , Chapman SA , Muench U. Evaluation of Clinical and Economic Outcomes Following Implementation of a Medicare Pay-for-Performance Program for Surgical Procedures. JAMA Netw Open 2021;4:e2121115 34406402 10.1001/jamanetworkopen.2021.21115 PMC8374611

[40] Swanson E. National Hospital Flash Report: Novermber 2024 Metrics. 2024.

[41] Pines JM , Isserman JA , Hinfey PB. The measurement of time to first antibiotic dose for pneumonia in the emergency department: a white paper and position statement prepared for the American Academy of Emergency Medicine. J Emerg Med 2009;37:335–340 19717266 10.1016/j.jemermed.2009.06.127

[42] Rhee C , Yu T , Wang R , et al. Association Between Implementation of the Severe Sepsis and Septic Shock Early Management Bundle Performance Measure and Outcomes in Patients With Suspected Sepsis in US Hospitals. JAMA Netw Open 2021;4:e2138596 34928358 10.1001/jamanetworkopen.2021.38596 PMC8689388

[43] Pakyz AL , Orndahl CM , Johns A , et al. Impact of the Centers for Medicare and Medicaid Services Sepsis Core Measure on Antibiotic Use. Clin Infect Dis 2021;72:556–565 32827032 10.1093/cid/ciaa456

[44] Anderson DJ , Moehring RW , Parish A , et al. The Impact of Centers for Medicare & Medicaid Services SEP-1 Core Measure Implementation on Antibacterial Utilization: A Retrospective Multicenter Longitudinal Cohort Study With Interrupted Time-Series Analysis. Clin Infect Dis 2022;75:503–511 34739080 10.1093/cid/ciab937

[45] National Quality Forum (NQF). Hospital Onset-Bacteremia and Fungemia Playbook. Washington, DC 2024.

[46] Bearman G , Doll M , Cooper K , Stevens MP. Hospital Infection Prevention: How Much Can We Prevent and How Hard Should We Try? Curr Infect Dis Rep 2019;21:2 30710181 10.1007/s11908-019-0660-2

[47] Hsu HE , Wang R , Broadwell C , et al. Association Between Federal Value-Based Incentive Programs and Health Care-Associated Infection Rates in Safety-Net and Non-Safety-Net Hospitals. JAMA Netw Open 2020;3:e209700 32639568 10.1001/jamanetworkopen.2020.9700 PMC7344380

[48] Blot K , Hammami N , Blot S , Vogelaers D , Lambert ML. Seasonal variation of hospital-acquired bloodstream infections: A national cohort study. Infect Control Hosp Epidemiol 2022;43:205–211 33975668 10.1017/ice.2021.85

[49] Giacobbe DR , Battaglini D , Ball L , et al. Bloodstream infections in critically ill patients with COVID-19. Eur J Clin Invest 2020;50:e13319 32535894 10.1111/eci.13319 PMC7323143

[50] Lubkin A , Bernard-Raichon L , DuMont AL , et al. SARS-CoV-2 infection predisposes patients to coinfection with Staphylococcus aureus. mBio 2024;15:e0166724 39037272 10.1128/mbio.01667-24 PMC11323729

[51] Horowitz HW. Infection control II: A practical guide to getting to zero. Am J Infect Control 2016;44:1075–1077 27158090 10.1016/j.ajic.2016.02.032

[52] Shively NR , Morgan DJ. The CDC antimicrobial use measure is not ready for public reporting or value-based programs. Antimicrob Steward Healthc Epidemiol 2023;3:e77 37113208 10.1017/ash.2023.143 PMC10127230

[53] Rhee C , Strich JR , Chiotos K , et al. Improving Sepsis Outcomes in the Era of Pay-for-Performance and Electronic Quality Measures: A Joint IDSA/ACEP/PIDS/SHEA/SHM/SIDP Position Paper. Clin Infect Dis 2024;78:505–513 37831591 10.1093/cid/ciad447 PMC11487102

[54] Centers for Disease Control and Prevention. 2022 National and State Healthcare-Associated Infections Progress Report. 2023. https://www.cdc.gov/healthcare-associated-infections/media/pdfs/2022-Progress-Report-Executive-Summary-H.pdf.

[55] Yu KC , Jung M , Ai C. Characteristics , costs, and outcomes associated with central-line-associated bloodstream infection and hospital-onset bacteremia and fungemia in US hospitals. Infect Control Hosp Epidemiol 2023;44:1920–1926 37424226 10.1017/ice.2023.132 PMC10755163

[56] de Kraker ME , Wolkewitz M , Davey PG , et al. Clinical impact of antimicrobial resistance in European hospitals: excess mortality and length of hospital stay related to methicillin-resistant Staphylococcus aureus bloodstream infections. Antimicrob Agents Chemother 2011;55:1598–1605 21220533 10.1128/AAC.01157-10 PMC3067153

[57] Agency for Healthcare Research and Quality. Estimating the Additional Hospital Inpatient Cost and Mortality Associated With Selected Hospital-Acquired Conditions. https://www.ahrq.gov/hai/pfp/haccost2017-results.html.

[58] Smith DRM , Pouwels KB , Hopkins S , Naylor NR , Smieszek T , Robotham JV. Epidemiology and health-economic burden of urinary-catheter-associated infection in English NHS hospitals: a probabilistic modelling study. J Hosp Infect 2019;103:44–54 31047934 10.1016/j.jhin.2019.04.010

[59] Nelson RE , Hatfield KM , Wolford H , et al. National Estimates of Healthcare Costs Associated With Multidrug-Resistant Bacterial Infections Among Hospitalized Patients in the United States. Clin Infect Dis 2021;72:S17–S26 33512523 10.1093/cid/ciaa1581 PMC11864165

[60] Umscheid CA , Mitchell MD , Doshi JA , Agarwal R , Williams K , Brennan PJ. Estimating the proportion of healthcare-associated infections that are reasonably preventable and the related mortality and costs. Infect Control Hosp Epidemiol 2011;32:101–114 21460463 10.1086/657912

[61] Rodriguez-Bano J , Garcia L , Ramirez E , et al. Long-term control of endemic hospital-wide methicillin-resistant Staphylococcus aureus (MRSA): the impact of targeted active surveillance for MRSA in patients and healthcare workers. Infect Control Hosp Epidemiol 2010;31:786–795 20524852 10.1086/654003

